# Frailty assessment based on trunk kinematic parameters during walking

**DOI:** 10.1186/s12984-015-0040-6

**Published:** 2015-05-24

**Authors:** Alicia Martínez-Ramírez, Ion Martinikorena, Marisol Gómez, Pablo Lecumberri, Nora Millor, Leocadio Rodríguez-Mañas, Francisco José García García, Mikel Izquierdo

**Affiliations:** Mathematics Department, Public University of Navarra, Campus de Arrosadía, 31006 Pamplona, Navarra Spain; Division of Geriatric Medicine, University Hospital of Getafe, Madrid, Spain; Division of Geriatric Medicine, Hospital Virgen del Valle, Complejo Hospitalario de Toledo, Toledo, Spain; Department of Health Sciences, Public University of Navarra, Pamplona, Navarra 31008 Spain

**Keywords:** Gait Analysis, Frailty, Accelerometer, Gyroscope, Inertial Sensors

## Abstract

**Background:**

Physical frailty has become the center of attention of basic, clinical and demographic research due to its incidence level and gravity of adverse outcomes with age. Frailty syndrome is estimated to affect 20 % of the population older than 75 years. Thus, one of the greatest current challenges in this field is to identify parameters that can discriminate between vulnerable and robust subjects. Gait analysis has been widely used to predict frailty. The aim of the present study was to investigate whether a collection of parameters extracted from the trunk acceleration signals could provide additional accurate information about frailty syndrome.

**Methods:**

A total of 718 subjects from an elderly population (319 males, 399 females; age: 75.4 ± 6.1 years, mass: 71.8 ± 12.4 kg, height: 158 ± 6 cm) volunteered to participate in this study. The subjects completed a 3-m walk test at their own gait velocity. Kinematic data were acquired from a tri-axial inertial orientation tracker.

**Findings:**

The spatio-temporal and frequency parameters measured in this study with an inertial sensor are related to gait disorders and showed significant differences among groups (frail, pre-frail and robust). A selection of those parameters improves frailty classification obtained to gait velocity, compared to classification model based on gait velocity solely.

**Interpretation:**

Gait parameters simultaneously used with gait velocity are able to provide useful information for a more accurate frailty classification. Moreover, this technique could improve the early detection of pre-frail status, allowing clinicians to perform measurements outside of a laboratory environment with the potential to prescribe a treatment for reversing their physical decline.

## Background

Interest in aging has grown exponentially over the last few decades. Some of the aspects of aging, such as disability and frailty, have become the focus of basic and clinical research [1–3]. Frailty syndrome is estimated to affect 20 % of the 75-year-old major population. The consequences of frailty range from the loss of mobility, which affects dependence, to institutionalization, hospitalization and death [3–5]. Moreover, the onset of frailty can be anticipated, avoided or delayed [1]. Under this framework, frailty reflects physiological deficiencies in organic systems, as well as in physical and cognitive function [6]. Consequently, affected individuals are more vulnerable to adverse events, such as falls, institutionalization and death [6].

Although gait disorders are not an inevitable part of aging, dysfunctions in balance and gait are common in old people due to musculo-skeletal, vascular and neurological disorders. Unlike young adults, older individuals exhibit deficiencies in mobility and gait that may result from multiple faults in different physiological systems [7]. Thus, the early detection of gait disorders has been demonstrated to be effective in identifying subclinical disability states [8], pathology detection [8] and fall risk prediction [9]. Moreover, this detection would provide an opportunity to reduce the functional drop [10].

In the area of mobility, gait and balance are fundamental scoreboards of lower extremity function and of a patient’s aptitude to carry out the activities of daily living (ADL) [11]. Movement limitations are the major reason for the loss of independence [12]. A decrease in the habitual gait velocity can be considered a predictor of serious consequences for the individual, including hospitalization or death. Nevertheless, in order to identify other characteristics of walking performance, gait analysis should not be limited to measurements of velocity.

Currently, an important research area linked to gait analysis is being developed to investigate the markers of frailty or other ailments and to improve the diagnosis of these conditions [13–15]. With this aim, different technologies that range from the rudimentary, including the slightly precise but cheap method of direct observation during walking, to complex, including expensive but accurate optoelectronic systems and force platforms, have been used [16]. Force platforms and optoelectronic systems are the gold standard for gait analysis, but their use remains restricted to the laboratory and is not readily applicable for clinical or domiciliary studies [17–19].

More recently, the use of inertial sensors has emerged as a promising alternative for analyzing human movements. Recent studies have demonstrated that the parameters obtained with these sensors during walking are significantly related to different motor deficiencies, frailty, Parkinson’s disorder or other diseases, such as diabetes and mild cognitive impairment [20–22].

The aim of this work was to examine the acceleration signals obtained from a tri-axial inertial magnetic sensor and to extract a collection of parameters to supply additional accurate information about frailty syndrome in a population of advanced age. We hypothesize that these parameters are able to provide useful information for detecting gait impairments and that this technique can provide helpful complementary information to identify frail populations.

## Methods

### Subjects

A total of 718 subjects from an elderly population (319 males, 399 females; age: 75.4 ± 6.1 years, mass: 71.8 ± 12.4 kg, height: 158 ± 6 cm) volunteered to participate in this study (Table 1).

**Table 1:** Subjects’ characteristics (Mean ± Std.)

	Robust	Pre-frail	Frail
	(n = 326)	(n = 327)	(n = 65)
Age (years)	73.4 ± 5.5	76.5 ± 5.6	80.2 ± 5.6
Female	183	178	38
Male	143	149	27
Height (cm)	158.3 ± 7.8	157.1 ± 9.2	155.7 ± 8.2
Body Mass Index	28.9 ± 4.1	28.9 ± 4.8	29.5 ± 5.1

We selected all of the subjects with available acceleration signals from the Toledo Study for Healthy Aging (TSHA). The TSHA is a Spanish longitudinal study designed to assess and to study frailty syndrome. Briefly, the TSHA comprises two population cohorts. The first cohort is formed by the survivors of the Toledo Study (a population-based cohort initiated in 1994), a population of men and women aged 77 years or older in 2006 [23]. The second cohort is formed by individuals between 65 and 76 years of age specifically recruited for this study in 2006. All people were subjected to the same assessment [24]. The study protocol was approved by the Clinical Research Ethics Committee of the Hospital Complex of Toledo, Spain. All study participants provided signed informed consent prior to their inclusion in the cohort.

Frailty was assessed using Fried’s criteria [6], but with the following adaptations: Weight loss was considered to be more than 4.5 kg of unintentional weight loss in the previous year. Slowness was defined using the walking speed test: individuals were asked to walk at their usual pace, following a standardized protocol; the slowest quintile was considered positive for this criterion. Exhaustion was assessed using two questions (“I felt that anything I did was a big effort” and “I felt that I could not keep on doing things”); the answers were scored between 0 and 4. This criterion was considered positive if any question was answered with a score of 2 or higher. Weakness was measured by grip strength using a Jamar hydraulic dynamometer in the dominant hand; the result was adjusted according to the subject’s body mass index. Subjects in the bottom quintile were considered positive for this criterion. Low physical activity was based on the Physical Activity Scale for the Elderly [25]; the worse quintile was considered positive for this criterion.

The subjects were classified as robust if any single criterion was met, as pre-frail if one or two criteria were met and frail if three or more of these criteria were met [6]. The exclusion criteria were any kind of leg diseases, shuffling gait, other degenerative diseases, or the inability to understand instructions or the questionnaire.

### Testing procedures

#### Walking test

The subjects walked in a straight line, without obstacles and at a self-regulated speed. The measurements were taken over a 3-m section, starting and ending limits were marked on the floor with tape lines leaving the first and last meters for acceleration and deceleration phases, respectively.

### Instrumentation

The walking test was performed with an inertial sensor, MTx (XSENS, Xsens Technologies B.V. Enschede, Netherlands), attached over the lumbar spine to record acceleration data. The MTx sensor combines nine individual MEMS sensors to provide drift-free 3D orientation, as well as kinematic data: 3D acceleration, 3D rate of turn (rate gyro) and 3D magnetometry.

The acceleration signal consists of gravitational and inertial components. The accelerometer registers gravity as a static vertical component, in addition to the dynamic acceleration caused by changes in velocity during locomotion. The gravity component must be subtracted to estimate the dynamic acceleration. The 3D orientation data provide the position of the inertial unit with respect to the gravitational vector, allowing the calculation of the inertial component for each axis. The gravitational constant was estimated by leaving the sensor still on a flat surface for two seconds.

To extract representative gait features, the signal intervals corresponding to the subject’s movement must be identified and separated. This task was performed by automatic peak detection. The first and last significant peaks of the signal were considered to mark the start and end of the movement, respectively.

A step was set as the interval between two peaks of the vertical acceleration component, corresponding to the moment of foot contact with the ground. To do this analysis based on the clipped signal, an exhaustive selection of the signal peaks was performed to determine the steps taken. To eliminate unrepresentative peaks and facilitate the identification of the most prominent ones, an approximation was made using a wavelet (Coif5 level 3) decomposition. The step pattern for all components of the signal, was calculated by taking the mean across all steps.

### Acceleration parameters

The following measured spatio-temporal parameters are found in the literature to be related to gait disorders [15, 22, 26–29]: step and stride regularity, gait symmetry, coefficient of variation (CoV) of the step time, signal root mean square (RMS) value and approximate entropy (ApEn). The frequential parameters analyzed in this study were the harmonic ratio (HR) and total harmonic distortion (THD). Each step period from every signal was obtained with single peak detection in the vertical acceleration signal. The rest of these parameters were obtained for three directions: vertical (VT), medio-lateral (ML) and antero-posterior (AP).

Step and stride regularity and gait symmetry were obtained from the autocorrelation sequence of the acceleration signal *x*. The autocorrelation coefficients *Am* for time lags *m* are defined as:

1 $$ {A}_m=\frac{1}{N-\left|m\right|}{\displaystyle {\sum}_{i= \max \left(0,-m\right)}^{\min \left(N-1,N-m-1\right)}{x}_i{x}_{i+m}}\kern2.25em m=-\left(N-1\right),\dots, N-1 $$

The estimation of step and stride regularity was performed by measuring the prominence of the first, *Ad1*, and second, *Ad2*, peaks after the central (zero lag) peak. Gait symmetry was computed through the difference between both peaks normalized to their maximum value.

2 $$ Sym=\frac{\left| Ad1- Ad2\right|}{ \max \left( Ad1, Ad2\right)} $$

Gait variability can be estimated by calculating the CoV of step time, where $\overline{t}$ is the mean of the step time across all steps and σ its standard deviation.

3 $$ CoV=\frac{\sigma }{\overline{t}} $$

The RMS value is defined by the following equation:

4 $$ RMS=\sqrt[2]{\frac{{\displaystyle {\sum}_{i=1}^N}{x}_i^2}{N}} $$

In statistics, ApEn is used to quantify the amount of regularity and the unpredictability of fluctuations in time-series data. The algorithm used to calculate ApEn for each signal was proposed by Ho *et al*. [30]. The frequential parameter HR was calculated by dividing the sum of the amplitude of the odd harmonics by that of the even harmonics, and the THD is the ratio between the sum of the amplitudes of all harmonics and the amplitude at the fundamental frequency.

5 $$ HR=\frac{{\displaystyle {\sum}_{i=0}^N}{A}_{2i+1}}{{\displaystyle {\sum}_{i=1}^{N+1}}{A}_{2i}} $$

Where “A” is the amplitude of the harmonics of the Fourier transform of the acceleration signal.

6 $$ THD=\frac{{\displaystyle {\sum}_{i=1}^N}{A}_i}{A_0} $$

In both cases, the first 20 harmonics were evaluated.

### Statistical analysis

Standard statistical methods were used for the calculation of the means and standard deviations (SD). The differences between the three groups (frail, pre-frail and control) were determined using one-way analysis of variance (ANOVA), with Newman-Keuls post hoc comparisons. When normality test failed (*p* < 0.05), Kruskal-Wallis One Way Analysis of Variance on Ranks test was used. The *p* < 0.05 criterion was used for establishing statistical significance. Dunn’s multiple comparison post hoc test was used to assess multiple comparisons. 95 % confidence intervals (95 % CI) were also calculated for each parameter.

A preliminary classification tree for the discrimination of frailty was used to determine the most relevant parameters from the set defined in this study. The importance of a variable was defined as the increase in prediction error when its values were permuted across the out-of-bag observations.

Then we defined two classification tree models to discriminate frailty. The first one uses only the gait velocity as discriminating measure. The second one uses the gait velocity and the previously obtained selection of relevant gait parameters. Both models were evaluated using the sensitivity, accuracy, specificity and precision for each frailty status.

The predictive accuracy of frailty of both models (gait velocity with and without gait parameters), was compared using receiver operating characteristic (ROC) curves analyses. Areas under the ROC curves (AUC) of the models were compared using the method of DeLong *et al*. [31].

## Results

### Gait analysis results and groups comparisons

**Fig. 1:**
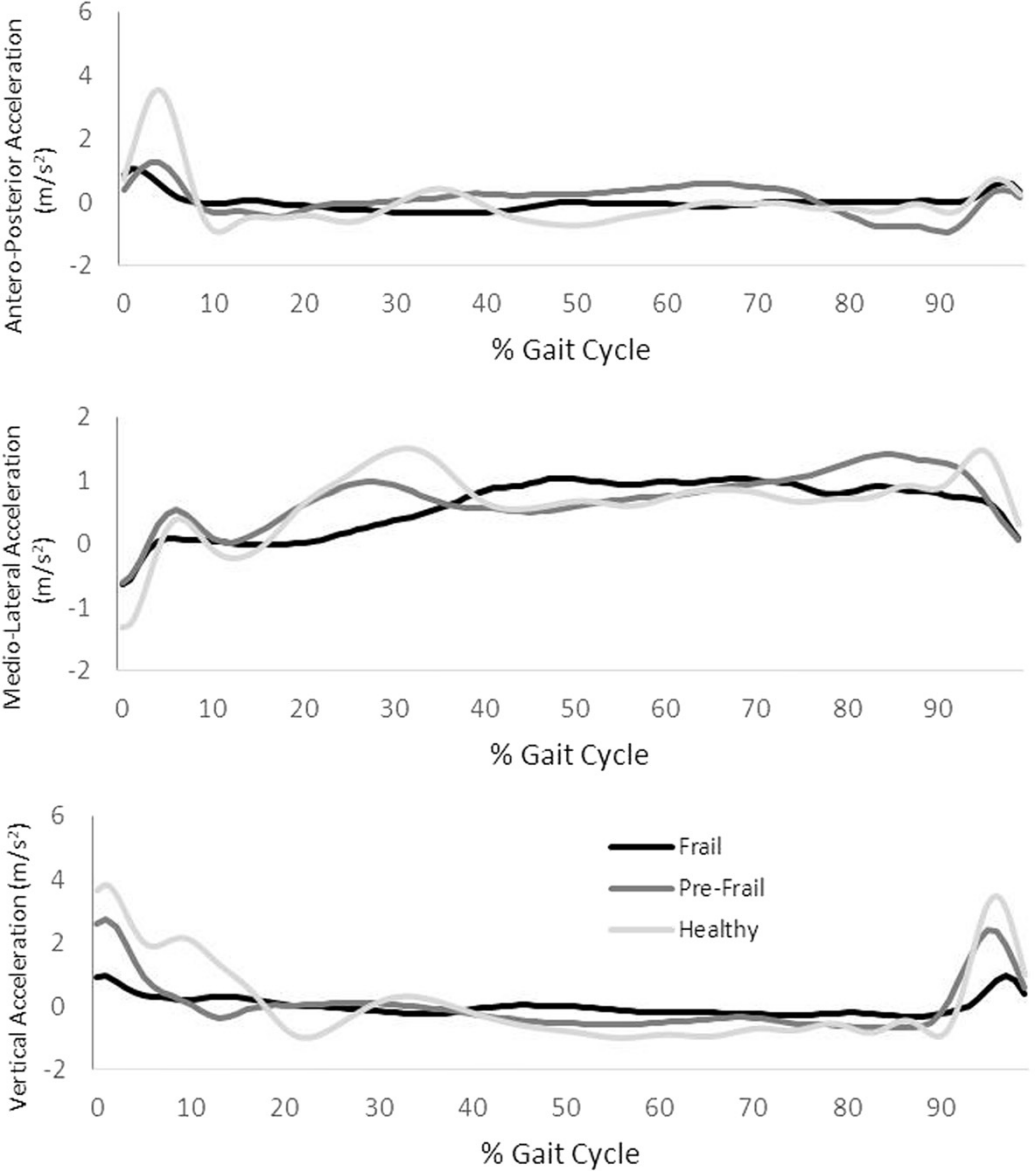
Mean antero-posterior, medio-lateral and vertical accelerations over multiple steps for one subject of each group (frail, pre-frail and robust)

Fig. 1 shows the mean of the antero-posterior, medio-lateral and vertical accelerations throughout the recorded steps for one subject of each group (frail, pre-frail and robust).

Table 2 shows the mean and standard deviations of parameter values in the VT, AP and ML directions. For all parameters measured in the VT direction, we observed significant differences (*p* < 0.05) between the three groups. In the AP component, significant differences were found in the RMS parameter (*p* < 0.05) between pre-frail and frail groups and between robust and frail; in contrast, we did not find any statistically significant differences in the other parameters among groups. In the ML component, significant differences were observed between groups only for the symmetry parameter (*p* < 0.05) and only between robust and frail groups. Since VT direction is the only component that shows significant differences between three groups, for the following statistical analysis and frailty classification analysis we will only consider this component of the signal.

**Table 2:** Mean parameter values, with standard deviations, in the VT, AP and ML directions and *p*-values between groups

		Robust	Pre-frail	Frail	*Statistical Significance*		
		Mean ± std.	Mean ± std.	Mean ± std.	R-PF	PF-F	R-F
Gait velocity (m/s)		0.59 ± 0.14	0.45 ± 0.13	0.32 ± 0.10	*	*	*
Cadence (step/min)		87.0 ± 14.8	88.4 ± 15.4	85.6 ± 16.6			
Step Regularity	AP	0.43 ± 0.16	0.40 ± 0.16	0.39 ± 0.17			
	ML	0.67 ± 0.16	0.69 ± 0.16	0.71 ± 0.14			
	VT	0.68 ± 0.16	0.58 ± 0.19	0.42 ± 0.21	*	*	*
Stride Regularity	AP	0.45 ± 0.17	0.40 ± 0.16	0.41 ± 0.17	*		
	ML	0.64 ± 0.18	0.64 ± 0.19	0.65 ± 0.16			
	VT	0.64 ± 0.17	0.58 ± 0.19	0.44 ± 0.21	*	*	*
Symmetry	AP	0.25 ± 0.20	0.24 ± 0.18	0.27 ± 0.19			
	ML	0.17 ± 0.15	0.16 ± 0.15	0.11 ± 0.11			*
	VT	0.14 ± 0.11	0.18 ± 0.15	0.25 ± 0.21	*	*	*
RMS	AP	1.00 ± 0.36	0.91 ± 0.33	0.77 ± 0.25		*	*
	ML	1.10 ± 0.31	1.15 ± 0.39	1.10 ± 0.29			
	VT	1.29 ± 0.44	1.04 ± 0.41	0.69 ± 0.29	*	*	*
Step Time CoV		0.10 ± 0.07	0.12 ± 0.08	0.15 ± 0.08	*	*	*
HR	AP	1.99 ± 0.60	1.96 ± 0.57	1.93 ± 0.61			
	ML	2.16 ± 0.64	2.09 ± 0.59	2.30 ± 0.83			
	VT	2.33 ± 0.55	2.15 ± 0.46	1.96 ± 0.57	*	*	*
THD	AP	3.61 ± 1.95	3.62 ± 2.02	3.56 ± 1.77			
	ML	1.60 ± 0.70	1.71 ± 0.88	1.60 ± 0.69			
	VT	2.68 ± 1.11	3.35 ± 1.64	4.43 ± 2.34	*	*	*
ApEn	AP	−0.63 ± 0.11	−0.66 ± 0.12	−0.62 ± 0.71			
	ML	−1.32 ± 2.91	−1.42 ± 4.17	−2.44 ± 8.93			
	VT	0.98 ± 2.19	1.96 ± 2.64	4.58 ± 3.85	*	*	*

*p < 0.05Acronyms: *Reg* regularity, *Sym* Symmetry, *RMS* signal root mean square value, *CoV* coefficient of variation of the step time, *ApEn* approximate entropy, *HR* harmonic ratio; total harmonic distortion (THD). All these parameters were obtained for three directions: vertical (VT), medio-lateral (ML) and antero-posterior (AP)

**Fig. 2:**
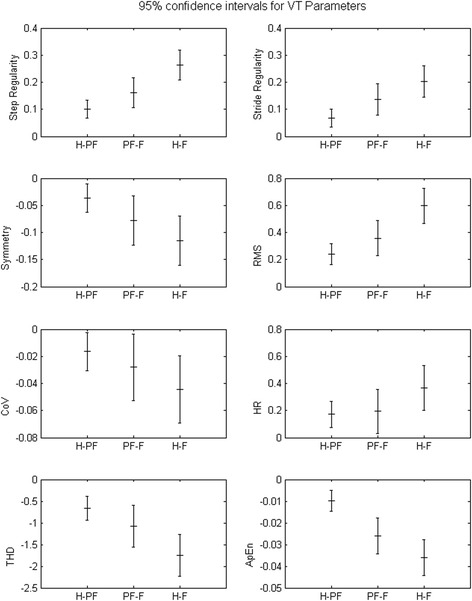
95 % Confidence Intervals (CI) for the difference of means between three groups are shown for all parameters measured in VT direction

Fig. 2 shows the 95 % CI of means differences for comparisons for the three groups for the VT component of each gait parameter.

### Frailty prediction and parameter selection

**Fig. 3:**
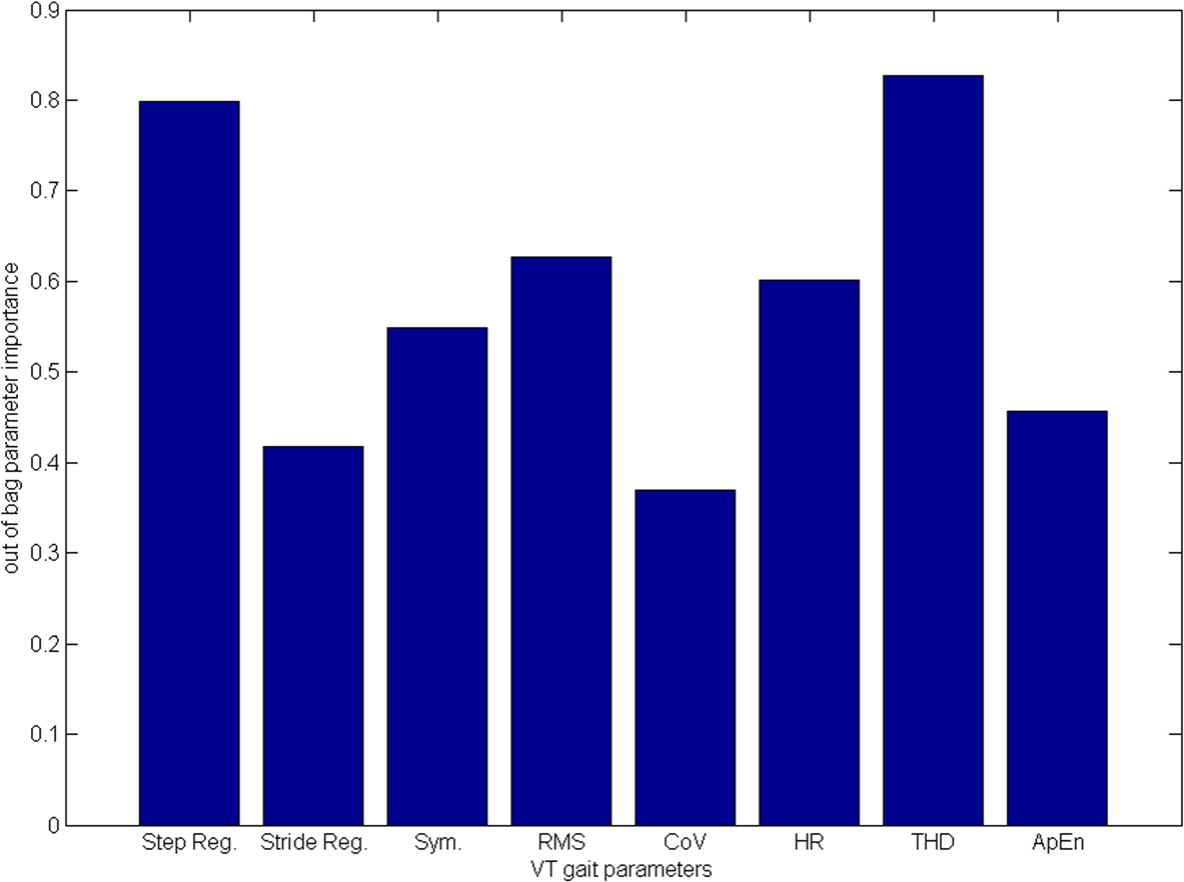
Decision Tree model to identify selected gait parameters

A decision tree model was used to identify the most appropriate gait parameters to discriminate the three groups. Fig. 3 shows the importance of each gait parameters defined in this study. It can be seen that the Step Regularity, the RMS and the THD have the highest scores among gait parameters. These three parameters were selected as the most appropriate to discriminate among robust, pre-frail and frail groups. We defined two classification tree models to discriminate frailty. The first one uses only the gait velocity as discriminating measure. Then the second model was defined using the gait velocity, the step regularity, the RMS and the THD.

To evaluate the usefulness of trunk accelerometry for the prediction of frailty, the sensitivity, specificity, accuracy and precision of both models (gait velocity with and without the selected gait parameters) were computed. They are shown in Table 3.

**Table 3:** Classification performance

		Sensitivity	Specificity	Accuracy	Precision
Gait Velocity	Robust	0.60	0.74	0.69	0.53
	Pre-Frail	0.18	0.68	0.52	0.23
	Frail	0.71	0.82	0.78	0.67
G. V. + Selected gait parameters	Robust	0.74	0.81	0.78	0.66
	Pre-Frail	0.48	0.78	0.68	0.53
	Frail	0.77	0.90	0.86	0.79

As we can see in the Table 3, the sensitivity, the specificity, the accuracy and the precision are significantly higher for the model with the selected gait parameters than for the model without the gait parameters.

**Fig. 4:**
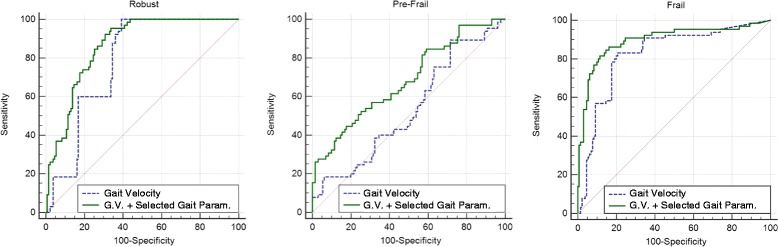
ROC curves of the two classificatory performance

The classification results for both models are shown in Table 4. In this table, AUC of both models for robust, pre-frail and frail groups is compared. As can be seen, gait velocity (AUC = 0.782; 0.535; 0.823), was less sensitive than gait velocity and selected gait parameters (AUC = 0.863; 0.683; 0.896) for the identification of robust, pre-frail and frail levels respectively. Fig. 4 shows the obtained ROC curves for the two classification models.

**Table 4:** Area under the curve comparison

	Gait Velocity	G. V. + Selected gait parameters	Pairwise comparisons of ROC curves		
			Difference between areas		
	AUC (95 %CI) ^a^	AUC (95 %CI) ^a^	Diff. ± S.E.^b^	95 % CI	*p*-Value
Robust	0.782 (0.717 – 0.838)	0.863 (0.807 – 0.908)	0.081 ± 0.028	0.025 - 0.137	0.004
Pre-Frail	0.535 (0.462 – 0.606)	0.683 (0.612 – 0.747)	0.148 ± 0.067	0.016 - 0.280	0.028
Frail	0.823 (0.762 – 0.874)	0.896 (0.844 – 0.935)	0.073 ± 0.019	0.037 - 0.110	<0.001

^a^ Binomial exact ^b^ DeLong *et al*.; *S.E.* Standard Error

## Discussion

In this study, we can conclude that, during walking, the parameters calculated from the vertical component of the acceleration signals from a body fixed sensor (i.e., one including a tri-axis accelerometer, magnetometer and gyroscope) may be of great interest in the clinical assessment of frailty syndrome. In fact, adding the proposed selection of gait parameters (Step Regularity, RMS value and THD), there is an improvement in frailty classification obtained to gait velocity, compared to classification model based on gait velocity solely. This result proves that there is a close relation between frailty and erratic gait patterns, beyond a slow gait, and it could give relevant information for frailty assessment.

Indeed, this improvement in frailty classification using the information provided by the signals obtained with an inertial sensor, is observed for the three study groups: robust, frail and pre-frail. Both sensitivity and specificity of the classification are improved and also AUC comparisons from ROC curves show significant differences for the three groups. Especially relevant is the case of the pre-frail group. The sensitivity for this group increases from 0.18 for the gait velocity classification to 0.48 when gait parameters are added to the model. Moreover, the overall performance of the classifier as measured with the AUC of the ROC curve, shows an increase from 0.53, equivalent to a random selector, to a value of 0.68. Pre-frail status has been described as a previous state of frailty decline where subjects have an increased probability of developing this syndrome [6], but also as a reversible state where an early detection of the symptoms and a suitable treatment could restore functional capacity [32]. Thus, a technique that could provide relevant information for pre-frail status detection would be of great importance.

As mentioned above, gait parameters obtained from the VT component of the acceleration signal are more determinant in discriminating frailty than those obtained from AP and ML directions. Indeed, from the AP direction, only the RMS value shows significant differences between robust and frail groups and also between pre-frail and frail groups. In the same way, for the ML direction only gait symmetry showed significant differences between robust and frail groups. Those results are in concordance with previous studies analyzing gait in impaired populations. It has been observed that regularity and symmetry of step and stride measured from trunk acceleration differ between groups with diverse mobility disorders and can predict adverse effects, such as falls or diseases such as Parkinson [22, 33]. In our study, the regularity of step and stride measured in the VT direction exhibited significant differences between the frail, pre-frail and robust groups.

The RMS value showed significant differences between the study groups in the AP and VT directions. The values obtained for the robust group were significantly higher than those for the pre-frail and frail group. In several studies, high RMS values for the acceleration signal have been associated with more intense movements in a person with greater motor control, which implies a reduced tendency toward future adverse events resulting from frailty [22]. This is clearly observed in the pattern of acceleration for each group. Furthermore, a low gait velocity, which is broadly associated with frailty, relates to a low signal level in acceleration patterns [34].

The approximate entropy or ApEn parameter also clearly differed between groups. As mentioned in the methods section, ApEn measures the predictability of consecutive steps, with a lower value corresponding to more regular series, consisting in blocks similar to one another, and higher values to chaotic series. Therefore, a regular gait pattern with similar consecutive steps would result in low values of ApEn, while higher values would indicate an irregular motion. This seems consistent with the results obtained for the aforementioned regularity parameters; the control group showed lower ApEn values than the pre-frail and frail groups. Therefore, this result reinforces the idea that the kinematic parameters obtained by trunk accelerometry during walking are valid identifiers of motor disorders [35] related to frailty syndrome. Moreover, since the majority of significant differences arise from the analysis of the VT component, we can deduce that the irregularities resulting from an erratic gait pattern manifest themselves through vertical movements.

The variability of parameters such as step length, step width, cadence and time support obtained from classic systems of gait analysis [14, 36–38] has been considered to be a marker of motor dysfunctions resulting from frailty and also predictors of adverse events. A recent study by Osaka *et al*. [16] found a strong correlation between these parameters and other gait parameters obtained with force platforms that have been widely accepted in gait analysis. Therefore, given the correlation between measurements obtained using force platforms and the variability of the step period obtained by trunk accelerometry during gait analysis, this feature may also serve as a good marker of frailty and adverse consequences. Our results are in agreement with the outcomes obtained by Osaka *et al*. [16] using force-platforms; they highlighted regularity and symmetry in the VT component and the RMS value as valid gait descriptors. In contrast, our results are obtained from an affordable and portable inertial unit.

Previous studies indicate that older people with stability problems exhibit lower HR values than individuals without these problems [15, 39]. This finding is related to our results, which indicated a greater HR in the control group. The HR is used as an indicator of smoothness and rhythm during walking [15]. The calculation of the harmonic ratio is based on the idea that the stride frequency, which is equivalent to two steps, is the fundamental gait frequency [29, 34]. Therefore, acceleration patterns that do not occur in multiples of two are considered “out of phase” and are indicative of irregular walking [34, 39].

Taking into account the frequency parameters, the THD is often used in signal processing to characterize the linearity of audio systems, power systems and radio communication systems. However, it has only recently been applied to gait analysis.

The frail group exhibited a higher THD value than the control group. As previously mentioned, the idea conveyed by this finding is that there is a greater presence of higher frequency components with respect to the fundamental gait in the frail group. Therefore, this result for the frail group suggests greater energy expenditure during gait corrections (fast movements) than during the performance of actual walking steps. By contrast, the control group expended most of their energy in the fundamental gait frequency, with less important contributions from the other frequency components. Consequently, a low THD level could indicate most of the energy expenditure performing the gait cycle would be focused in the stride movement, which suggests a regular and efficient gait pattern.

Summarizing, the frail group exhibited less “intense” movements with fewer motor control and more irregular series during walking than the robust and pre-frail groups, Moreover, the frail group exhibited reduced similarity with erratic gait pattern mainly through vertical movements, and greater energy expenditure in gait corrections.

An important limitation of the present study needs to be mentioned. Although in our study we have included elderly people that were able to walk, many frail elderly subjects exhibit shuffling gait. Their inability to perform stepping movements restricts the use of tri-axial inertial sensors with the purpose of gait analysis in this specific population. However, the aim of our study is to find subtle differences among groups that would otherwise go unnoticed and to investigate the differences in gait patterns among people that are able to walk. In the future, subsequent studies will be needed to investigate how frailty status and these walking parameters relate to important consequences, such as hospitalization and death.

## Conclusion

We can conclude that the proposed selection of walking parameters (Step Regularity, RMS and THD) are able to demonstrate differences among individuals of varying frailty status and that simultaneously used with gait velocity measures, can provide useful information from an erratic walk for a more accurate frailty classification. Particularly in the case of pre-frail subjects this technique could improve the early detection of this state, allowing the clinician to prescribe a treatment for reversing their physical decline. Thus, this simple and portable technique provides a useful clinical methodology for assessing frailty in ambulatory environments.

## Abbreviations

ADL: Activities of daily living
TSHA: Toledo study for healthy aging
CoV: Coefficient of variation
RMS: Root mean square
ApEn: Approximate entropy
HR: Harmonic ratio
THD: Total harmonic distortion
VT: Vertical
AP: Antero-posterior
ML: Medio-lateral
SD: Standard deviations
ANOVA: Analysis of variance
CI: Confidence intervals
ROC: Receiver operating characteristic
AUC: Areas under the curve

## References

[1] Morley JE (2008). Diabetes, Sarcopenia, and Frailty. Clin Geriatr Med.

[2] Janssen I, Heymsfield SB, Ross R (2002). Low Relative Skeletal Muscle Mass (Sarcopenia) in Older Persons Is Associated with Functional Impairment and Physical Disability. J Am Geriatr Soc.

[3] Guralnik JM, Ferrucci L, Simonsick EM, Salive ME, Wallace RB (1995). Lower-Extremity Function in Persons over the Age of 70 Years as a Predictor of Subsequent Disability. N Engl J Med.

[4] Hogan DB, MacKnight C, Bergman H, Steering Committee, Canadian Initiative on Frailty and Aging (2003). Models, definitions, and criteria of frailty. Aging Clin Exp Res.

[5] Visser M, Deeg DJ, Lips P, Longitudinal Aging Study Amsterdam (2003). Low vitamin D and high parathyroid hormone levels as determinants of loss of muscle strength and muscle mass (sarcopenia): the Longitudinal Aging Study Amsterdam. J Clin Endocrinol Metab.

[6] Fried LP, Tangen CM, Walston J, Newman AB, Hirsch C, Gottdiener J, Seeman T, Tracy R, Kop WJ, Burke G, McBurnie MA (2001). Frailty in Older Adults: Evidence for a Phenotype. J Gerontol A: Biol Med Sci.

[7] Hausdorff JM and NB Alexander. Gait disorders: Evaluation and Management. 6000 Broken Sound Parkway NW, Suite 300, Boca Raton 7: CRC Press. 2005.

[8] Studenski S, Perera S, Wallace D, Chandler JM, Duncan PW, Rooney E, Fox M, Guralnik JM (2003). Physical Performance Measures in the Clinical Setting. J Am Geriatr Soc.

[9] Montero-Odasso M, Schapira M, Duque G, Soriano E, Kaplan R, Camera L (2005). Gait disorders are associated with non-cardiovascular falls in elderly people: a preliminary study. BMC Geriatr.

[10] Cesari M, Kritchevsky SB, Penninx BWHJ, Nicklas BJ, Simonsick EM, Newman AB, Tylavsky FA, Brach JS, Satterfield S, Bauer DC, Visser M, Rubin SM, Harris TB, Pahor M (2005). Prognostic Value of Usual Gait Speed in Well-Functioning Older People?Results from the Health, Aging and Body Composition Study. J Am Geriatr Soc.

[11] Berg K, Norman KE (1996). Functional assessment of balance and gait. Clin Geriatr Med.

[12] Tinetti ME, Williams TF, Mayewski R (1986). Fall risk index for elderly patients based on number of chronic disabilities. Am J Med.

[13] Purser JL, Kuchibhatla MN, Fillenbaum GG, Harding T, Peterson ED, Alexander KP (2006). Identifying Frailty in Hospitalized Older Adults with Significant Coronary Artery Disease. J Am Geriatr Soc.

[14] Callisaya ML, Blizzard L, Schmidt MD, McGinley JL, Srikanth VK (2010). Ageing and gait variability—a population-based study of older people. Age Ageing.

[15] Menz HB, Lord SR, Fitzpatrick RC (2003). Age-related differences in walking stability. Age Ageing.

[16] Osaka H, Shinkoda K, Watanabe S, Fujita D, Ishida H, Kobara K, Yoshimura Y, Ito T (2013). Validity of Evaluation Index Utilizing Three Components of Trunk Acceleration during Walking. J Phys Ther Sci.

[17] Alaqtash M, Sarkodie-Gyan T, Yu H, Fuentes O, Brower R, and Abdelgawad A. Automatic classification of pathological gait patterns using ground reaction forces and machine learning algorithms. In: 33rd Annual International Conference of the IEEE Engineering in Medicine and Biology Society (EMBC): 2011. 453-457. 10.1109/IEMBS.2011.6090063 22254346

[18] Bergmann G, Deuretzbacher G, Heller M, Graichen F, Rohlmann A, Strauss J, Duda GN (2001). Hip contact forces and gait patterns from routine activities. J Biomech.

[19] Horváth M, Tihanyi T, Tihanyi J (2001). Kinematic and kinetic analyses of gait patterns in hemiplegic patients. Facta Univ Series: Phys Educ Sport.

[20] Millor N, Lecumberri P, Gomez M, Martinez-Ramirez A, Izquierdo M (2013). An evaluation of the 30-s chair stand test in older adults: frailty detection based on kinematic parameters from a single inertial unit. J Neuroeng Rehabil.

[21] Martínez-Ramírez A, Lecumberri P, Gómez M, Rodriguez-Mañas L, García FJ, Izquierdo M (2011). Frailty assessment based on wavelet analysis during quiet standing balance test. J Biomech.

[22] Yang CC, Hsu YL, Shih KS, Lu JM (2011). Real-time gait cycle parameter recognition using a wearable accelerometry system. Sensors (Basel).

[23] García Garcíaa FJ, Sánchez Ayalab MI, Martín Correaa E, Marsal Alonsoc C, Rodríguez Ferrera G, Colmenerod CG, Romero Rizosa L, Rodríguez Barqueroa MJ (2001). Prevalencia de demencia y de sus subtipos principales en sujetos mayores de 65 ań os: efecto de la educación y ocupación. Estudio Toledo. Med Clin.

[24] Garcia-Garcia FJ, Gutierrez Avila G, Alfaro-Acha A, Amor Andres MS, Escribano Aparicio MV, Humanes Aparicio S, Larrion Zugasti JL, Gomez-Serranillo Reus M, Rodriguez-Artalejo F, Rodriguez-Manas L, De Los Angeles De La Torre Lanza M, Toledo Study Group (2011). The prevalence of frailty syndrome in an older population from Spain. The Toledo Study for Healthy Aging. J Nutr Health Aging.

[25] Schuit AJ, Schouten EG, Westerterp KR, Saris WH (1997). Validity of the Physical Activity Scale for the Elderly (PASE): according to energy expenditure assessed by the doubly labeled water method. J Clin Epidemiol.

[26] Moe-Nilssen R, Helbostad JL (2004). Estimation of gait cycle characteristics by trunk accelerometry. J Biomech.

[27] Karmakar CK, Khandoker AH, Begg RK, Palaniswami M, and Taylor S. Understanding Ageing Effects by Approximate Entropy Analysis of gait variability. In: 29th Annual International Conference of the IEEE Engineering in Medicine and Biology Society (EMBC): 2007. 1965-1968. 10.1109/IEMBS.2007.4352703 18002369

[28] Montero-Odasso M, Muir SW, Hall M, Doherty TJ, Kloseck M, Beauchet O, Speechley M (2011). Gait Variability Is Associated With Frailty in Community-dwelling Older Adults. J Gerontol A: Biol Med Sci.

[29] Brach JS, McGurl D, Wert D, VanSwearingen JM, Perera S, Cham R, Studenski S (2011). Validation of a Measure of Smoothness of Walking. J Gerontol A: Biol Med Sci.

[30] Ho KKL, Moody GB, Peng C, Mietus JE, Larson MG, Levy D, Goldberger AL (1997). Predicting Survival in Heart Failure Case and Control Subjects by Use of Fully Automated Methods for Deriving Nonlinear and Conventional Indices of Heart Rate Dynamics. Circulation.

[31] DeLong ER, DeLong DM, Clarke-Pearson DL (1988). Comparing the areas under two or more correlated receiver operating characteristic curves: a nonparametric approach. Biometrics.

[32] Greene BR, Doheny EP, O'Halloran A, Anne Kenny R (2014). Frailty status can be accurately assessed using inertial sensors and the TUG test. Age Ageing.

[33] Yang M, Zheng H, Wang H, McClean S, Hall J, Harris N (2010). “Assessing accelerometer based gait features to support gait analysis for people with Complex Regional Pain Syndrome.”. Proceedings of the 3rd International Conference on PErvasive Technologies Related to Assistive EnvironmentsAnonymous : Association for Computing Machinery (ACM).

[34] Menz HB, Lord SR, Fitzpatrick RC (2003). Acceleration patterns of the head and pelvis when walking on level and irregular surfaces. Gait Posture.

[35] Hamacher D, Singh NB, Van Dieen JH, Heller MO, Taylor WR (2011). Kinematic measures for assessing gait stability in elderly individuals: a systematic review. J R Soc Interface.

[36] Moe-Nilssen R, Helbostad JL (2005). Interstride trunk acceleration variability but not step width variability can differentiate between fit and frail older adults. Gait Posture.

[37] Tao W, Liu T, Zheng R, Feng H (2012). Gait Analysis Using Wearable Sensors. Sensors.

[38] Hausdorff J (2005). Gait variability: methods, modeling and meaning. J NeuroEngineering Rehab.

[39] Yack HJ, Berger RC (1993). Dynamic Stability in the Elderly: Identifying a Possible Measure. J Gerontol.

